# Supra‐SAC: Need and Role for an All‐of‐Government Scientific Advisory Committee

**DOI:** 10.1002/gch2.201700075

**Published:** 2018-09-27

**Authors:** John‐Arne Røttingen, Trygve Ottersen

**Affiliations:** ^1^ The Research Council of Norway P.O. Box 564 1327 Lysaker Norway; ^2^ Harvard T.H. Chan School of Public Health Harvard University Boston MA 02115 USA; ^3^ Division for Health Services Norwegian Institute of Public Health P.O. Box 4404 Nydalen 0403 Oslo Norway; ^4^ Oslo Group on Global Health Policy Department of Community Medicine and Centre for Global Health University of Oslo P.O. Box 1130, Blindern 0318 Oslo Norway

Put crudely, there are two schools of thought when it comes to how to mobilize the global evidence base to better inform public policy. One approach is to let the evidence speak for itself, or in other words, systematically review and synthesize research evidence on the specific question or topic and then provide a succinct summary of what the evidence shows to policy makers. Another approach is to assemble a committee of scientists or experts, which may be called a scientific advisory committee (SAC), and then get them to assess the evidence and provide advice to guide policy options. In general, there seems to be a trend towards more of the first and less of the latter which strengthens the validity and opportunity for scrutiny through more transparency. However, many of the difficult and complex policy questions need a combination of both systematic reviews and deliberative processes. Which questions to formulate and study are often scientific discussions in their own right, and the total body of knowledge gives often equivocal answers which needs further studies and debates. Scientific deliberations within SACs is therefore often a crucial part of a system well equipped to provide evidence‐informed policy making.

Several countries have established SACs and similar mechanisms for science advice to inform policy making. The UK has a long standing chief scientific advisor with departmental science advisers for the different sectoral ministries. New Zealand has a similar system with a vocal and active chief science advisor who has also been instrumental in creating the International Network for Government Science Advice. The United States has an Office of Science and Technology Policy with its head normally also co‐chairing the President's Council of Advisors on Science and Technology. The European Union has had individuals as chief scientists, but has now established a high‐level group at the core of its revised scientific advice mechanism. The experiences with these and similar arrangements have recently been summarized elsewhere,^1^ and several countries are now in process of instituting formal scientific advice mechanisms to inform policy making or are considering the options for doing so. Canada is recruiting its first chief science advisor, and in Norway there is an ongoing discussion of what would be the most useful roles for a potential new national council to support evidence‐informed policy.


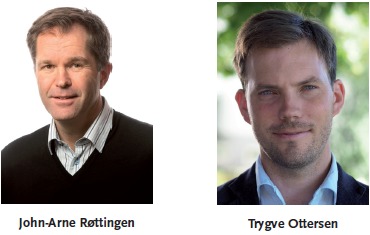


**Figure 1:**
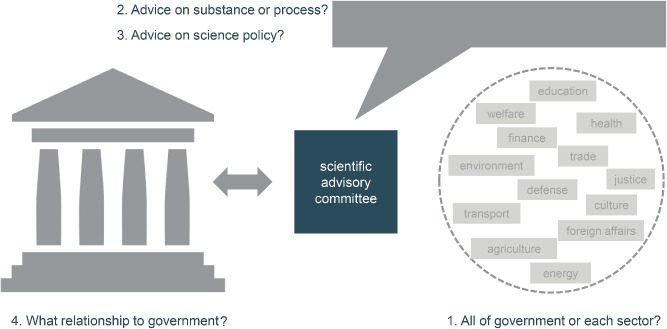
Four key questions when establishing a systematic approach to science advice.

When a government is considering establishing a systematic approach to science advice, there are four key questions that need to be addressed, as illustrated in **Figure** **1**. Should there be specific SACs set up for each sector and ministry, one common mechanism for all sectors, or some combination? Should the SACs provide input on specific substantive issues, on the policy process—e.g., how to use and integrate research evidence in policy making—or some combination? What should be the role of the SACs in policy for science? What should be the relationship between government and the mechanisms set up?

First, the question of whether most sectors need to establish their own science advice mechanisms or whether an all‐of‐government mechanism is sufficient and more efficient needs to be considered. Many countries have strong science advice functions on economic policy, public health, food safety, environmental issues, security and energy, and some countries have established similar mechanisms on education, welfare services, criminal justice, and development aid. Given the different areas of expertise required to address the important questions in the different sectors, and also the number of issues that can benefit from a scientific assessment, there is a case for separate mechanisms. However, many of the current grand societal challenges are multisectorial and require transdisciplinary insights and analyses. Important examples are climate change mitigation and adaption and larger societal transitions like digitalization. This argues for a joint all‐of‐government mechanism—a supra SAC—to avoid sectorial lock ins and that individual SACs only assess the issue at stake from their perspective. It is also the case that the various sectors often face the same challenges in establishing and developing a system for science advice, and a cross‐sectorial mechanism can facilitate mutual learning. An all‐of‐government mechanism could thus be of service to all ministries and departments.

Second, one needs to decide on whether the science advice mechanisms should be engaged in specific substantive issues, i.e., policy content, or rather work at a meta‐level, i.e., policy processes. To analyze and assess concrete scientific issues requires sufficient insights and expertise and a strong secretariat function that can prepare research syntheses and other documents for the advisor or the advisory committee. This may be demanding. Another approach is to only play an oversight function in a system of smaller and specific and possibly ad hoc SACs and other mechanisms for evidence‐informed policy making. Such a system role and stewardship function can be carried out by setting standards and defining norms and principles, and by monitoring the practice of policy making and whether norms are met. This function related to the processes of policy making could be served by recommending governmental guidelines for how research should be utilized and by examining how research and scientific and technological insights have been built upon in governmental and parliamentary policy papers and processes.

Third, the roles and responsibilities of SACs need to be thought through. Most will agree that the core function is about giving advice to policy (“science for policy”), either on process or content. However, this is also somewhat linked to giving advice on science policy (“policy for science”). A process‐related role is linked to the function of advocating for and demonstrating the value of research in society and promoting public engagement in science, whereas a content related role often will identify research gaps and thereby needs for more or new research. Both of those functions are generally seen as responsibilities for a research‐policy mechanism which can be linked to research funding or be an integral part of a research funding agency. The interplay and interface between science for policy and policy for science mechanisms therefore needs to be considered.

Fourth, the formal relationship between science‐advice mechanisms and government needs to be carefully considered. How independent or dependent should the SACs or similar mechanisms be? Most will strongly argue that they need to be independent in their operations. In other words, they should have full autonomy when it comes to the way they work and the conclusions they make. Such degrees of freedom are important to secure legitimacy. At the same time, they need to get their mandate and work program from government—for supra‐SACs possibly at the cabinet level—to secure political buy in. In this sense, they therefore should be fully dependent or reliant of the state. If not, they will not have sufficient links to policy and politics, and can easily become irrelevant or just outside commentators. Striking this balance is not easy and requires careful judgment and opportunities for building trust and relationships. Such governmental mechanisms therefore play a different role than academic or learned societies and scientific academies that are fully independent, and should therefore also find a good way of working with and involving such organizations. In Europe, the scientific advice mechanism has indeed established a method of working with the SAPEA, the Science Advice for Policy by European Academies consortium.

The answers to these four questions need to be tailored to the national context. However, we believe the case for some more general answers can be made. We would argue for specific scientific advice mechanisms being established within each sector or subsector, guided by need and complexity of the issues. At the same time, we believe there is a need for a supra‐SAC directly linked to the prime minister's office or equivalent that can take an all‐of‐government and all‐sectors approach. We believe such a supra‐SAC should first and foremost focus on the policy process and only provide substantive advice on the most crucial multi‐sectorial issues. The supra‐SAC should complement, facilitate and work together with the sector‐based advisory functions, as well as the institutional mechanisms responsible for advising on science policy. SACs of this kind should be clearly owned by the government, while being secured sufficient autonomy in its operations. Overall, we see great advantages in a supra‐SAC that is superficial on content and broad on process, that complements sector‐specific mechanisms and advisory mechanisms for science policy, and that is independent from government while depending on it.
